# Supplementary material for: Green CURIOCITY: a study protocol for a European birth cohort study analysing childhood heat-related health impacts and protective effects of urban natural environments
Source: BMJ Open. 2022 Jan 24;12(1):e052537. doi: 10.1136/bmjopen-2021-052537 (PMC8788192; doi:10.1136/bmjopen-2021-052537)
Supplement: Author's manuscript [file bmjopen-2021-052537.draft_revisions.pdf]

BMJ Open is committed to open peer review. As part of this commitment we make the peer review history of every article we publish publicly available.

When an article is published we post the peer reviewers' comments and the authors' responses online. We also post the versions of the paper that were used during peer review. These are the versions that the peer review comments apply to.

The versions of the paper that follow are the versions that were submitted during the peer review process. They are not the versions of record or the final published versions. They should not be cited or distributed as the published version of this manuscript.

BMJ Open is an open access journal and the full, final, typeset and author-corrected version of record of the manuscript is available on our site with no access controls, subscription charges or pay-per-view fees (http://bmjopen.bmj.com).

If you have any questions on BMJ Open's open peer review process please email info.bmjopen@bmj.com

BMJ Open

Green CURIOCITY: A study protocol for a European birth cohort study analysing childhood heat-related health impacts and protective effects of urban natural environments.

Journal:	*BMJ Open*
Manuscript ID	bmjopen-2021-052537
Article Type:	Protocol
Date Submitted by the Author:	20-Apr-2021

Complete List of Authors:	van den Bosch, Matilda; Barcelona Institute for Global Health; Universitat Pompeu Fabra<br>Basagaña, Xavier; Barcelona Institute for Global Health; Universitat Pompeu Fabra<br>Mudu, Pierpaolo; World Health Organization European Centre for Environment and Health<br>Kendrovski, Vladimir; World Health Organization European Centre for Environment and Health<br>Maitre, Léa; Barcelona Institute for Global Health<br>Hjertager Krog, Norun; Norwegian Institute of Public Health<br>Aasvang, Gunn Marit; Norwegian Institute of Public Health<br>Grazuleviciene, Regina; Vytauto Didziojo Universitetas<br>McEachan, Rosemary; Bradford Institute for Health Research<br>Vrijheid, Martine; Barcelona Institute for Global Health; Universitat Pompeu Fabra<br>Nieuwenhuijsen, Mark; Barcelona Institute for Global Health; Universitat Pompeu Fabra

Keywords:	EPIDEMIOLOGY, PREVENTIVE MEDICINE, PUBLIC HEALTH

SCHOLARONE™
Manuscripts

TITLE PAGE

Title: **Green CURIOCITY: A study protocol for a European birth cohort study analysing childhood heat-related health impacts and protective effects of urban natural environments.**

Authors: van den Bosch, Matilda[1,2,3,4,5], Basagaña, Xavier[1,2,3], Mudu, Pierpaolo[6], Kendrovski, Vladimir[6], Maitre, Léa[1,2,3], Krog, Norun Hjertager[7], Aasvang, Gunn Marit[7], Grazuleviciene, Regina[8], McEachan, Rosemary[9], Vrijheid, Martine[1,2,3], Nieuwenhuijsen, Mark[1,2,3]

Corresponding author: Matilda van den Bosch, Barcelona Institute for Global Health, Doctor Aiguader, 88, 08003 Barcelona, Spain; matilda.vandenbosch@isglobal.org; +34-631827093

[1]ISGlobal, Barcelona, Spain

[2]Universitat Pompeu Fabra (UPF), Barcelona, Spain

[3]CIBER Epidemiología y Salud Pública (CIBERESP), Madrid, Spain

[4]School of Population and Public Health, The University of British Columbia, Vancouver, Canada

[5]Department of Forest and Conservation Sciences, The University of British Columbia, Vancouver, Canada

[6]World Health Organization European Centre for Environment and Health, Bonn, Germany

[7]Norwegian Institute of Public Health, Oslo, Norway

[8]Vytauto Didziojo Universitetas, Kaunas, Lithuania

[9]Bradford Institute for Health Research, Bradford, United Kingdom

Keywords: Childhood health, heat exposure, green spaces, climate change, urban planning

Word count: 2991

For peer review only - http://bmjopen.bmj.com/site/about/guidelines.xhtml

ABSTRACT

Introduction. The European climate is getting warmer and the impact on childhood health and development is insufficiently understood. Equally, how heat related health risks can be reduced through nature-based solutions (NBS), such as exposure to urban natural environments, is unknown. Green CURe In Outdoor CITY spaces (Green CURIOCITY) will analyse how heat exposure during pregnancy affects birth outcomes and how long-term heat exposure may influence children's neurodevelopment. We will also investigate if adverse effects can be mitigated by urban natural environments. A final goal is to visualise intraurban patterns of heat vulnerability and assist planning towards healthier cities.

Methods and analysis. We will use existing data from the Human Early-Life Exposure (HELIX) cohort, which includes information on birth outcomes and neurodevelopment from six European birth cohorts. The cohort is linked to data on prenatal heat exposure and impact on birth outcomes will be analysed with logistic regression models, adjusting for air pollution and noise and socio-behavioural co-variates. Similarly, impact of cumulative and immediate heat exposure on neurodevelopmental outcomes at age five will be assessed. For both analyses, the potentially moderating impact of natural environments will be quantified. For visualisation, Geographic Information Systems (GIS) data will be combined to develop vulnerability maps, demonstrating urban "hot-spots" where the risk of negative impacts of heat is aggravated due to socio-demographic and land use patterns. Finally, geospatial and meteorological data will be used for informing GreenUr, an existing software prototype developed by the WHO Regional Office for Europe to quantify health impacts and augment policy tools for urban green space planning.

Ethics and dissemination. The protocol was approved by the Comité Ético de Investigación Clínica Parc de Salut MAR, Spain. Findings will be published in peer-reviewed journals and presented at policy events. Through stakeholder engagement, the results will also reach user groups and practitioners

For peer review only

STRENGTHS AND LIMITATIONS OF THIS STUDY

- The project will use harmonised data from a large pan-European cohort enabling assessments across different climate zones and
- The accessibility of long-term temporal exposure data facilitates analyses of vulnerability windows
- Analyses of participants who have changed residence over the study period will partly address self-selection bias
- The development of visualisation and policy tools in Green CURIOCITY will provide opportunities for immediate implementation and impact on healthy urban planning
- Some of the cohort contains a disproportionately high number of participants from the highest educational level, potentially limiting the representativeness of the results

For peer review only - http://bmjopen.bmj.com/site/about/guidelines.xhtml

INTRODUCTION

The European climate is getting warmer and the summer of 2019 experienced multiple severe heat waves, shattering all-time temperature records in cities across the continent (1). The morbidity and mortality toll of these events is yet to be unfolded, but we already know the dire effects on a number of vulnerable populations, including more than 20 children who became seriously ill after experiencing temperatures above 40°C in the Netherlands (2). Following climate change, Europe can expect higher average daily temperatures and more frequent heatwaves (3) with severe health and economic consequences (4). The forecast is even grimmer in cities than in rural areas due to the urban heat island effect (5) and long-term effects are insufficiently understood. Heat also aggravates negative impacts of air pollution (6) and intensifies symptoms and mortality risk of many chronic diseases (7, 8). Many countries are unprepared to fully address this challenge, and some populations are at higher risks than others due to, for example, socioeconomic (9), behavioural (10), or demographic factors (11) that reduce resilience. Understanding the full extent of climate change related health impacts across the life course is imperative to accurately act and address the issue, through mitigation, adaption, and treatment options that are available for everyone.

Children are particularly vulnerable to both acute and long-term heat exposure due to a constellation of biological and behavioural factors, including physiology, metabolism, and activity patterns (12, 13). This increases the risk of heat-related morbidity and negative impacts. Recent reviews and research (14, 15) suggests that also prenatal heat exposure has negative effects, possibly because of an increase in oxidative stress and systemic inflammation in response to temperature changes during pregnancy (16, 17). However, additional evidence is clearly needed due to the paucity of studies that have examined the effects of long-term heat exposure on birth outcomes and childhood development. Prior research has largely focused on adults and little is known about how to reduce risks among children. To address this escalating threat, improved evidence and innovative solutions are urgently needed, including insights into risk differences due to sex, socioeconomic status (SES), and living context.

Nature-based solutions (NBS) – defined as “actions which are inspired by, supported by, or copied from nature” designed to address environmental challenges in an adaptive manner while providing economic, social, and environmental benefits (18) – offer unique potential for addressing this threat. The EU Research and Innovation Policy Agenda on NBS and Re-Naturing cities aims to position the EU as a leader in ‘innovating with nature’ for more resilient societies. In this context, urban natural environments (UNEs, comprising both green spaces such as parks and street trees and blue spaces such as rivers and lakes) stand out as effective NBS to reduce heat-related health risks by cooling via increased shade and evapotranspiration. Existing evidence suggests that the temperature-reducing effect of UNEs ranges from 0.4-3.0°C, or even more, depending on local context (19, 20), with even-larger effects during the night (21). Other, more technical, adaptation strategies exist, but many have proven to be less efficient (22) or energy-intensive (e.g. air condition) (23) consequently escalating greenhouse gas emissions and exacerbating climate change (24). The urgent need to identify more-sustainable solutions (including NBS) is recognised in a forthcoming *Lancet* series on Heat and Health (25).

In parallel, an increasing amount of research indicates numerous childhood health benefits from UNEs, including reduced prevalence of asthma (26), ADHD (27), and overweight (28), and improved birth outcomes (29) and cognitive and other neurodevelopmental functions (30)

For peer review only - http://bmjopen.bmj.com/site/about/guidelines.xhtml

with impact across the life course (31). Various pathways for these health benefits have been suggested, but evidence is inconsistent. From this perspective, the mitigating effect of UNEs on heat and subsequent health impacts should be further investigated. As an example, UNEs may contribute to a generally cooler climate, reducing health-related harms of long-term heat exposure. A systematic review from 2016 concluded that the cooling effect of UNEs is one of the more-likely pathways between UNEs and health and that research on this mediator should be prioritised (32).

Knowledge gaps around climate change, childhood health, and Nature Based Solutions

The full impact of the effects of climate change on children's health via acute and long-term heat exposure remains unclear, including differences between climate zones. To identify intra-urban inequalities and areas of greater vulnerability, the roles of specific land-use and socioeconomic factors merit further investigation. The potential of NBS to prevent heat-related health risks must also be analysed in more depth. Although the elderly and chronically ill also represent vulnerable populations, the focus on children is critical from a Developmental Origin of Health and Disease perspective (33), because prenatal and childhood exposures impact health across the life course. Cold-weather events also pose health risks (34), but climate change is more clearly linked to heat-related health risks (35). The research project Green CURe In Outdoor CITY spaces (Green CURIOCITY) will address these knowledge gaps and guide the implementation of sustainable and equitable solutions, including NBS, to address heat exposure and impaired childhood health and development. The topic is of high urgency and relevance because climate change will inevitably increase heat-related morbidity in vulnerable populations, including among preborn and children, and better understanding of NBS could be an approach to reduce current and future risks to health and society.

Aims and Objectives

The objective of Green CURIOCITY is to address the outlined knowledge gaps by analysing the association between pre- and postnatal heat exposure and childhood birth and neurodevelopmental outcomes in different European climate zones. Further on, the aim is to identify whether neighbourhood exposure to UNEs is associated with a lower risk of heat-related adverse health outcomes among children. The goal is also to address implementation by identifying and mapping spatial "hotspots" of childhood heat vulnerability in different European cities with the aim of visualising where interventions, such as NBS, should be prioritised. Finally, the project aims to influence policy making by contributing to further information to a plugin tool, GreenUr, for Geographic Information System (GIS) software (QGIS), developed by the WHO Regional Office for Europe. GreenUr measures availability and accessibility of green space and quantifies direct and indirect health impacts at a city scale and Green CURIOCITY aims to specifically contribute to opportunities for quantifying health benefits from urban green spaces within the context of heat reduction.

METHODS AND ANALYSIS

The inferential studies of Green CURIOCITY are based on linkages of environmental exposure data to cohort data from the Human Early-Life Exposome (HELIX) project (36). HELIX is a collaborative project of six ongoing, longitudinal, population-based birth cohort studies in Europe funded through the EU FP7 Exposome Research programme. Pregnant women in the original cohorts were recruited between 1999 and 2010, and all cohorts included at least one

For peer review only - http://bmjopen.bmj.com/site/about/guidelines.xhtml

follow-up during the prenatal period, at birth, and after delivery. The complete HELIX study population includes 31,472 mother-child pairs at the time of delivery and all information is stored in a common data warehouse. The harmonisation process of data from the national cohorts in HELIX followed expert input for standardisation rules and was based on a coding-system, subsequent to checking and matching of cohort-specific variables (36).

Study area and national cohorts

The birth cohorts of HELIX include the Born in Bradford (BiB) study in the UK (37), the Étude des Déterminants pré et postnatals du dévelopment et de la santé de l'Enfant (EDEN) study in France (38), the INfancia y Medio Ambiente (INMA) cohort in Spain (39), the Kaunus cohort (KANC) in Lithuania (40), the Norwegian Mother, Father and Child Cohort Study (MoBa) (41), and the RHEA Mother Child Cohort study in Crete, Greece (42) (Table 1).

Table 1 Summary of the national cohorts included in HELIX. Adapted from Maitre et al. (2018) (36)

Cohort	Years of birth	Region covered by HELIX	No. of births in HELIX entire cohort
**BiB, UK**	2007-2010	Bradford	10,849
**EDEN, France**	2003-2006	Nancy and Poitiers, urban areas	1,900
**INMA, Spain**	2003-2008	Gipuzkoa, Sabadell, Valencia	2,063
**KANC, Lithuania**	2007-2008	Kaunas	4,107
**MoBa, Norway**	1999-2008	Oslo	11,095
**RHEA, Greece**	2007-2008	Heraklion	1,458
**Total**			**31,472**

BiB: Born in Bradford; EDEN: Étude des Déterminants pré et postnatals du development et de la santé de l'Enfant; INMA: INfancia y Medio Ambiente; KANC: Kaunas cohort; MoBa: Norwegian Mother, Father and Child Cohort Study.

Primary outcomes in Green CURIOCITY: Birth outcome data are available for the entire cohort (n=31,472) and include gestational duration, birth weight and length, and size for gestational age. Exclusion criteria in most of the cohorts were twin pregnancies, women with diabetes or pregnancy induced hypertension, and mothers under 16 years of age.

The availability of neurodevelopmental indicators varies across the cohorts, but data have been harmonised for cognitive function (verbal and non-verbal) and for psychomotor function (fine and gross motor function). In total 11,423 children have harmonised data for cognitive and/or psychomotor function linked to environmental exposures. Not all cohorts collected data for all the different outcomes (Table 2). KANC did not collect data on neurodevelopmental outcomes.

Table 2. Harmonised neurodevelopmental outcome data per cohort to be used in Green CURIOCITY. (N=11,423)

Neurodevelopmental outcome	BiB	EDEN	INMA	MoBa	RHEA	Subjects in harmonised dataset
**Cognition function**	0	1,100	1,420	0	779	3,299
**Verbal cognitive function**	2,057	1,107	1,420	5,834	779	11,197
**Non-verbal cognitive function**	0	1,107	1,420	0	779	3,306
**Psychomotor function**	0	0	1,420	5,939	779	8,138
**Gross motor function**	0	0	1,420	5,864	779	8,112
**Fine motor function**	1,909	1,120	1,420	5,913	779	11,092

For peer review only - http://bmjopen.bmj.com/site/about/guidelines.xhtml

All outcome variables have been harmonised and standardised to a mean of 100 and a standard deviation of 15, making them comparable across the cohorts. The tests that were used for various neurodevelopmental outcomes in the respective cohorts are listed in table 3. All tests were evaluated at the age of 4.5 years.

Table 3. The respective tests and scales used in the separate cohorts before harmonisation.

	Cognitive function			Psychomotor function		
	Cognitive function	Verbal cognition	Non-verbal cognition	Psychomotor function	Gross motor	Fine motor
**BiB, UK**						
British Picture Vocabulary Scale (43)		x				
Clinical Kinematic Assessment Tool (44)						x
**EDEN, France**						
Weschler Preschool and Primary Scale of Intelligence (45)	x	x	x			
Peg moving task (46)						x
**INMA, Spain**						
McCarthy Scales of Children's Abilities (47)	X	X	X	X	X	X
**MoBa, Norway**						
Child Development Inventory (48)		X		X	X	X
**RHEA, Greece**						
McCarthy Scales of Children's Abilities (47)	x	x	x	x	x	x

Co-variates

The interactions between environmental exposures, childhood health outcomes and other social, biological, and cultural variables are complex and the evidence of associations is often inconsistent. The final selection of confounder variables to include in the analyses will be determined a priori based on existing evidence and by using a Directed Acyclic Graphs (DAG) approach. HELIX contains extensive information on a number of key covariates and based on a preliminary literature search on potential confounders of the association between prenatal heat exposure and birth outcomes, we will consider including child sex, season of birth (49, 50), urban living during pregnancy (51), maternal age and education (52, 53), maternal body mass index (BMI) (54), parental country of origin (55), maternal health behaviour (such as smoking and alcohol intake) during pregnancy (56), and individual and area level socioeconomic deprivation indicators (54). The analysis of neurodevelopmental outcomes will consider the same confounders and, in addition, maternal cognitive function (57) and attendance to nursery school (58).

Environmental exposure variables

For peer review only - http://bmjopen.bmj.com/site/about/guidelines.xhtml

Environmental exposure data for Green CURIOCITY exist in an integrated Geographic Information System (GIS) environment, contained in the HELIX database. Using complete residential address histories, exposure estimates per trimester during the prenatal period and average annual values up to age of neurodevelopmental assessment have been assigned to the participants and we will test different spatial analysis units.
A heat exposure index will be developed based on four variables: (1) temperature (average of mean/max/min during pregnancy period, per trimester, and at birth); (2) land surface temperature (LST, during pregnancy, per trimester, and at birth); (3) relative humidity (average of mean/max/min during pregnancy period, per trimester, and at birth); and (4) atmospheric pressure during pregnancy and per trimester. The index value per trimester will be linked to data on birth outcomes and the opportunity for assessing specific heat waves will be explored. Impact on neurodevelopment will be assessed by linking the heat index value prenatally as well as postnatal annual values up to the age of outcome assessment.

To assess the modifying impact of urban natural spaces on the association between heat exposure and childhood health outcomes, we will use data from the European Environmental Agency's 'Urban Atlas' (59), apart from for the Norwegian cohort (MoBa) where a similar local land cover map, Norwegian map, N50, was used (60). The Urban Atlas contains 17 land use classes and is developed from image classification and interpretation of high-resolution satellite imagery. The accuracy level is approximately 85% for artificial surfaces. Across European cities, it provides harmonised information of land use, including, for example, green urban areas, water, sport and recreational facilities, impervious surfaces, and built-up areas. Apart from exposure values, we will use area and distance to nearest green and blue spaces, as of previous protocols (61, 62). We will also use a general vegetation indicator, the Normalized Difference Vegetation Index (NDVI) (63), derived from 30m spatial resolution Landsat images and provided in raster maps. The values are averaged for buffers zones of 100, 300, and 500 m around residence. The project will adjust for air pollution exposure (NO_2, NOx, PM_{10}, $PM_{2.5}$) available from land use regression models (64). The values are extrapolated back in time, using data from existing regulatory monitors, and averaged per trimester, pregnancy period, birth, and annually. In addition, we will control for noise exposure, available from European road traffic noise maps with continuous and categorical values (L_{den}).

Statistical analysis

We will use logistic regression to estimate the association between trimester-specific temperature and risk of short gestational duration or small for gestational age and linear regression to estimate the association between trimester-specific temperature and term birth weight, standardized via z-scores and adjusted for key co-variates. Results will be pooled overall and by geographic regions and climate zones. In addition, stratified analyses will be conducted to assess moderation by sex and SES. We will develop structural equation models (SEMs) to explore the potential for UNEs to mitigate adverse heat-related effects on birth outcomes. SEM analyses will allow us to test the statistical significance of a mediation effect (i.e., that improved birth outcomes occur via cooling effects of UNEs), while adjusting for confounding and neighbourhood dependency. Using SEM will also allow us to detect indirect effects (i.e., that UNEs reduce harmful effects of heat even if direct heat-related effects on birth outcomes are not detected). Results will be pooled by climate zone and we will run sensitivity analyses adjusting for air pollution and noise. We will also develop logistic regression models to test the association between long-term heat exposure and neurodevelopment, adjusting for key confounders. We will apply time-weighted averages across the full exposure period, pre- and postnatally, carrying out sub-analyses among children

For peer review only - http://bmjopen.bmj.com/site/about/guidelines.xhtml

who have moved to assess the impact of exposure length and to partially address self-selection bias. Additional analyses will be stratified by sex and SES and pooled by geographic region and climate zone. We will use SEM with a similar approach and rationale as for the birth outcome analyses, assessing the mitigating effect of UNE exposure, as well as similar sensitivity analyses.

Mapping and spatial overlay design

For the "visualisation" study of Green CURIOCITY we will conduct a spatial overlay analysis to identify areas where different environmental (e.g. low amount of UNEs) or demographic (e.g. low age or low income) vulnerability factors coincide with high temperatures, so called urban "hotspots". Geospatial data capturing the intraurban spatial distribution of environmental (heat, UNE, built environment, and impervious surfaces) vulnerability indicators will be downloaded from the HELIX GIS environment and demographic data (age group, sex, and SES) from municipal resources. Environmental data will be averaged per the spatial unit that is aligned with SES data aggregation in respective cities. Subsequently, we will apply a ranking method and identify the most-vulnerable areas, based on prevalence of vulnerability factors, using a cut-off of 25%, similar to the approach used by Aminipouri et al. (2016) (65). The 25% threshold is an easily interpretable number, which makes the approach useful for city officials to replicate for making decisions about directing investments in NBS interventions.

Software expansion

For the implementation tool, expansion of an existing software prototype developed for QGIS by WHO (GreenUr, https://www.euro.who.int/en/health-topics/environment-and-health/urban-health/activities/greenur-the-green-urban-spaces-and-health-tool) will be developed based on adaptation of existing algorithms and modelled quantification of heat related health effects following changes in UNEs. Raster (Landsat) and vector data (Urban Atlas) of UNEs in combination with meteorological input data will be incorporated in the models.

PATIENT AND PUBLIC INVOLVEMENT Neither patients nor the public were involved in the development of this protocol. Patient and public involvement were, however, part of the wider HELIX project per each cohort.

ETHICS AND DISSEMINATION

All data to be used in Green CURIOCITY are available from the HELIX cohort. The six national cohorts on which HELIX is based had undergone ethical review and approval at initiation and new approvals were acquired for the work in HELIX. An additional evaluation and ethics approval has been acquired for Green CUROICTY project from the Comité Ético de investigación Clínica Parc de Salut MAR, Spain (reference number 2020/9567/I). The data are pseudonymised and all procedures of Green CURIOCITY that will include analyses of study participants will follow the ethics guidelines of ISGlobal's Ethics Committee and the HELIX Ethics Task Force (36). Data will be safeguarded to protect the confidentiality and anonymity of the participants and is consistent with the terms of the consent signed by participants in the respective national cohorts. Adequate measures to ensure health data protection and confidentiality will be taken, according to European directive on the protection of individuals with regard to the processing of personal data and free movement of such data (2016/679, General Data Protection Regulation, GDPR). All reported results will pertain to

For peer review only - http://bmjopen.bmj.com/site/about/guidelines.xhtml

analyses of aggregate data; no variables or combination of variables that can identify an individual will be associated with any published or unpublished report of the study.

Results from Green CURIOCITY will be disseminated through high-impact scientific journals, conference presentations, expert seminars, and policy briefs. The project has connections to broad international, interdisciplinary networks, such as the International Union for Conservation of Nature and the World Health Organization (WHO), which will support dissemination to a global audience. Methods and results will also be communicated through research briefs and reports and via institutional social media platforms and project websites. There are plans for roundtable events with urban planners and key decision-makers to share results of the vulnerability-mapping exercises and to integrate findings into sustainable urban plans, although these strategies may have to be adapted due to the current COVID-19 situation. In addition, the project aims to prepare educational material for distribution at schools, health care clinics, and public health websites, including infographics and worksheets describing how heat impacts children, summarizing recommendations regarding “green” schoolyards and playgrounds, and highlighting the importance of heat prevention during pregnancy.

CONCLUSION

Green CURIOCITY will be the first comprehensive, European-wide study to explore associations between heat exposure and both birth outcomes and childhood neurodevelopment and the modifying impact of urban natural spaces. It puts a strong emphasis on identifying intraurban differences in heat vulnerability and prevention to address nature-based solutions, specifically the cooling effects of UNEs. It will directly support sustainable policies, both by informing urban planning solutions and by strengthening the application of a GIS-based tool, developed by WHO/EURO, that aims at raising awareness and facilitating decision-making when considering green spaces because of their contribution to public health.

AUTHORS’ CONTRIBUTIONS

MvdB drafted this manuscript on the basis of a grant proposal that was devised and written by MvdB in collaboration with MN, XB, PM, and VK. MV is principal investigator of HELIX. LM coordinated the collection and harmonisation of the entire HELIX database. GMA, RG, RM co-ordinated HELIX data collection and research protocols in respective national cohorts. NHK provided environmental data for MoBa. All authors contributed to drafting, editing, and reviewing the manuscript. All authors read the final version of this manuscript, approved it for submission for publication, and agreed to be accountable for all aspects of the work. The proposal was approved for funding on 31/03/2020.

FUNDING STATEMENT

This work is supported by the European Commission through a Horizon 2020 Marie Skłodowska-Curie Grant, Reference number 891538.

LM is funded by a Juan de la Cierva-Incorporación fellowship (IJC2018-035394-I) awarded by the Spanish Ministerio de Economía, Industria y Competitividad.

For peer review only - http://bmjopen.bmj.com/site/about/guidelines.xhtml

COMPETING INTERESTS STATEMENT

Nothing to declare.

PM and VK are staff members of the WHO. The authors alone are responsible for the views expressed in this publication and they do not necessarily represent the decisions or stated policy of the WHO.

REFERENCES

1. Mitchell D, Kornhuber K, Huntingford C, Uhe P. The day the 2003 European heatwave record was broken. The Lancet Planetary Health. 2019;3(7):e290-e2.
2. APNews. The Latest: Heat wave smashes weather records in Europe 2019 [Available from: https://www.apnews.com/e29811558e0344e6ae0f326dadfab2e1.
3. Meehl GA, Tebaldi C. More intense, more frequent, and longer lasting heat waves in the 21st century. Science. 2004;305.
4. Peng RD, Bobb JF, Tebaldi C, McDaniel L, Bell ML, Dominici F. Toward a quantitative estimate of future heat wave mortality under global climate change. Environ Health Perspect. 2011;119(5):701-6.
5. Oke TR. City size and the urban heat island. Atmospheric Environment. 1973;7(8):769-79.
6. McCormack MC, Belli AJ, Waugh D, Matsui EC, Peng RD, Williams DL, et al. Respiratory effects of indoor heat and the interaction with air pollution in chronic obstructive pulmonary disease. Annals of the American Thoracic Society. 2016;13(12):2125-31.
7. Watts N, Amann M, Arnell N, Ayeb-Karlsson S, Belesova K, Berry H, et al. The 2018 report of the Lancet Countdown on health and climate change: shaping the health of nations for centuries to come. The Lancet. 2018;392(10163):2479-514.
8. Xu R, Zhao Q, Coelho MSZS, SaldivaPaulo H.N., Zoungas S, Huxley RR, et al. Association between Heat Exposure and Hospitalization for Diabetes in Brazil during 2000-2015: A Nationwide Case-Crossover Study. Environmental Health Perspectives. 2019;127(11):117005.
9. Reid CE, O'Neill MS, Gronlund CJ, Brines SJ, Brown DG, Diez-Roux AV, et al. Mapping community determinants of heat vulnerability. Environ Health Perspect. 2009;117(11):1730-6.
10. Johnson DP, Stanforth A, Lulla V, Luber G. Developing an applied extreme heat vulnerability index utilizing socioeconomic and environmental data. Applied Geography. 2012;35(1-2):23-31.
11. Díaz J, Carmona R, Mirón I, Ortiz C, León I, Linares C. Geographical variation in relative risks associated with heat: update of Spain's Heat Wave Prevention Plan. Environment international. 2015;85:273-83.
12. Xu Z, Etzel RA, Su H, Huang C, Guo Y, Tong S. Impact of ambient temperature on children's health: A systematic review. Environmental Research. 2012;117:120-31.
13. Xu Z, Sheffield PE, Su H, Wang X, Bi Y, Tong S. The impact of heat waves on children's health: a systematic review. International Journal of Biometeorology. 2014;58(2):239-47.
14. Zhang Y, Yu C, Wang L. Temperature exposure during pregnancy and birth outcomes: An updated systematic review of epidemiological evidence. Environmental Pollution. 2017;225:700-12.
15. Spolter F, Kloog I, Dorman M, Novack L, Erez O, Raz R. Prenatal exposure to ambient air temperature and risk of early delivery. Environ Int. 2020;142:105824.
16. Halonen JI, Zanobetti A, Sparrow D, Vokonas PS, Schwartz J. Associations between outdoor temperature and markers of inflammation: a cohort study. Environmental Health. 2010;9(1):42.

For peer review only - http://bmjopen.bmj.com/site/about/guidelines.xhtml

17. Ferguson KK, Kamai EM, Cantonwine DE, Mukherjee B, Meeker JD, McElrath TF. Associations between repeated ultrasound measures of fetal growth and biomarkers of maternal oxidative stress and inflammation in pregnancy. American Journal of Reproductive Immunology. 2018;80(4):e13017.

18. ECDG. Towards an EU Research and Innovation Policy Agenda for Nature-Based Solutions & Re-Naturing Cities Final Report of the Horizon 2020 Expert Group on "Nature-Based Solutions and Re-Naturing Cities". Luxembourg; 2015.

19. TNC. Planting healthy air: A global analysis of the role of urban trees in addressing particulate matter pollution and extreme heat. The Nature Conservancy; 2016.

20. Venter ZS, Krog NH, Barton DN. Linking green infrastructure to urban heat and human health risk mitigation in Oslo, Norway. Science of The Total Environment. 2020;709:136193.

21. Fryd O, Pauleit S, Bühler O. The role of urban green space and trees in relation to climate change. CAB Reviews: Perspectives in Agriculture, Veterinary Science, Nutrition and Natural Resources. 2011;6:1-18.

22. Weinberger KR, Zanobetti A, Schwartz J, Wellenius GA. Effectiveness of National Weather Service heat alerts in preventing mortality in 20 US cities. Environment International. 2018;116:30-8.

23. Davis LW, Gertler PJ. Contribution of air conditioning adoption to future energy use under global warming. Proceedings of the National Academy of Sciences. 2015;112(19):5962-7.

24. Abel DW, Holloway T, Harkey M, Meier P, Ahl D, Limaye VS, et al. Air-quality-related health impacts from climate change and from adaptation of cooling demand for buildings in the eastern United States: An interdisciplinary modeling study. PLoS Medicine. 2018;15(7).

25. Capon A, Jay O, Ebi K, Lo S. Heat and health: a forthcoming Lancet Series. The Lancet. 2019;394(10198):551-2.

26. Sbihi H, Tamburic L, Koehoorn M, Brauer M. Greenness and incident childhood asthma: A 10-year follow-up in a population-based birth cohort. American Journal of Respiratory and Critical Care Medicine. 2015;192(9):1131-3.

27. Donovan GH, Michael YL, Gatziolis D, Mannetje At, Douwes J. Association between exposure to the natural environment, rurality, and attention-deficit hyperactivity disorder in children in New Zealand: a linkage study. The Lancet Planetary Health. 2019;3(5):e226-e34.

28. Bao W-W, Yang B-Y, Zou Z-Y, Ma J, Jing J, Wang H-J, et al. Greenness surrounding schools and adiposity in children and adolescents: Findings from a national population-based study in China. Environmental Research. 2021;192:110289.

29. Akaraci S, Feng X, Suesse T, Jalaludin B, Astell-Burt T. A Systematic Review and Meta-Analysis of Associations between Green and Blue Spaces and Birth Outcomes. International journal of environmental research and public health. 2020;17(8):2949.

30. Mygind L, Kurtzhals M, Nowell C, Melby PS, Stevenson MP, Nieuwenhuijsen M, et al. Landscapes of becoming social: A systematic review of evidence for associations and pathways between interactions with nature and socioemotional development in children. Environment International. 2021;146:106238.

31. Browning MHEM, Rigolon A. Could nature help children rise out of poverty? Green space and future earnings from a cohort in ten U.S. cities. Environmental Research. 2019.

32. **van den Bosch** M, Ode Sang A. Urban natural environments as nature-based solutions for improved public health - A systematic review of reviews. Environ Res. 2017;158:373-84.

33. Gluckman P, Hanson M. The developmental origins of health and disease: An overview. Developmental Origins of Health and Disease2006. p. 1-5.

34. Gasparrini A, Guo Y, Hashizume M, Lavigne E, Zanobetti A, Schwartz J. Mortality risk attributable to high and low ambient temperature: a multicountry observational study. Lancet. 2015;386.

35. Gasparrini A, Guo Y, Sera F, Vicedo-Cabrera AM, Huber V, Tong S, et al. Projections of temperature-related excess mortality under climate change scenarios. The Lancet Planetary Health. 2017.

For peer review only - http://bmjopen.bmj.com/site/about/guidelines.xhtml

36. Maitre L, de Bont J, Casas M, Robinson O, Aasvang GM, Agier L, et al. Human Early Life Exposome (HELIX) study: a European population-based exposome cohort. BMJ Open. 2018;8(9):e021311.
37. Wright J, Small N, Raynor P, Tuffnell D, Bhopal R, Cameron N, et al. Cohort Profile: The Born in Bradford multi-ethnic family cohort study. International Journal of Epidemiology. 2012;42(4):978-91.
38. Heude B, Forhan A, Slama R, Douhaud L, Bedel S, Saurel-Cubizolles M-J, et al. Cohort Profile: The EDEN mother-child cohort on the prenatal and early postnatal determinants of child health and development. International Journal of Epidemiology. 2015;45(2):353-63.
39. Guxens M, Ballester F, Espada M, Fernández MF, Grimalt JO, Ibarluzea J, et al. Cohort profile: the INMA—INfancia y Medio Ambiente—(environment and childhood) project. International journal of epidemiology. 2012;41(4):930-40.
40. Grazuleviciene R, Danileviciute A, Nadisauskiene R, Vencloviene J. Maternal smoking, GSTM1 and GSTT1 polymorphism and susceptibility to adverse pregnancy outcomes. International journal of environmental research and public health. 2009;6(3):1282-97.
41. Magnus P, Birke C, Vejrup K, Haugan A, Alsaker E, Daltveit AK, et al. Cohort profile update: the Norwegian mother and child cohort study (MoBa). International journal of epidemiology. 2016;45(2):382-8.
42. Chatzi L, Leventakou V, Vafeiadi M, Koutra K, Roumeliotaki T, Chalkiadaki G, et al. Cohort profile: the mother-child cohort in Crete, Greece (Rhea study). International journal of epidemiology. 2017;46(5):1392-3k.
43. Dunn LM, Dunn DM. The British picture vocabulary scale: GL Assessment Limited; 2009.
44. Culmer PR, Levesley MC, Mon-Williams M, Williams JH. A new tool for assessing human movement: the Kinematic Assessment Tool. J Neurosci Methods. 2009;184(1):184-92.
45. Warschausky S. Wechsler Preschool and Primary Scale of Intelligence. In: Kreutzer JS, DeLuca J, Caplan B, editors. Encyclopedia of Clinical Neuropsychology. New York, NY: Springer New York; 2011. p. 2690-3.
46. Annett M. Handedness and Braisn Assymetry. 1 ed. London: Psychology Press; 2001.
47. Levin E. McCarthy Scales of Children's Abilities. In: Goldstein S, Naglieri JA, editors. Encyclopedia of Child Behavior and Development. Boston, MA: Springer US; 2011. p. 928-9.
48. Ireton H, Glascoe FP. Assessing children's development using parents' reports. The Child Development Inventory. Clin Pediatr (Phila). 1995;34(5):248-55.
49. Grootendorst-Van Mil NH, Steegers-Theunissen RPM, Hofman A, Jaddoe VWV, Verhulst FC, Tiemeier H. Brighter children? The association between seasonality of birth and child IQ in a population-based birth cohort. BMJ Open. 2017;7(2).
50. Hemati Z, Keikha M, Riahi R, Daniali SS, Goudarzi M, Kelishadi R. A systematic review on the association of month and season of birth with future anthropometric measures. Pediatric Research. 2020.
51. Skrivankova V, Zwahlen M, Adams M, Low N, Kuehni C, Egger M. Spatial epidemiology of gestational age and birth weight in Switzerland: Census-based linkage study. BMJ Open. 2019;9(10).
52. Shapiro GD, Bushnik T, Wilkins R, Kramer MS, Kaufman JS, Sheppard AJ, et al. Adverse birth outcomes in relation to maternal marital and cohabitation status in Canada. Annals of Epidemiology. 2018;28(8):503-9.e11.
53. Weng YH, Yang CY, Chiu YW. Risk assessment of adverse birth outcomes in relation to maternal age. PLoS ONE. 2014;9(12).
54. Clayborne ZM, Giesbrecht GF, Bell RC, Tomfohr-Madsen LM. Relations between neighbourhood socioeconomic status and birth outcomes are mediated by maternal weight. Social Science and Medicine. 2017;175:143-51.

For peer review only

55. Komro KA, Markowitz S, Livingston MD, Wagenaar AC. Effects of State-Level Earned Income Tax Credit Laws on Birth Outcomes by Race and Ethnicity. Health Equity. 2019;3(1):61-7.
56. Bird AL, Grant CC, Bandara DK, Mohal J, Atatoa-Carr P, Wise MR, et al. Maternal health in pregnancy and associations with adverse birth outcomes: Evidence from Growing Up in New Zealand. Aust New Zealand J Obstet Gynaecol. 2017;57(1):16-24.
57. Ronfani L, Vecchi Brumatti L, Mariuz M, Tognin V, Bin M, Ferluga V, et al. The complex interaction between home environment, socioeconomic status, maternal IQ and early child neurocognitive development: A multivariate analysis of data collected in a newborn cohort study. PLoS ONE. 2015;10(5).
58. Barnett WS. Long-Term Effects of Early Childhood Programs on Cognitive and School Outcomes. The Future of Children. 1995;5(3):25-50.
59. European_Union. Mapping Guide for a European Urban Atlas. European Environment Agency, EEA, Copenhagen, Denmark; 2011.
60. https://kartkatalog.geonorge.no/metadata/n50-map-data/ea192681-d039-42ec-b1bc-f3ce04c189ac. [
61. Robinson O, Tamayo I, de Castro M, Valentin A, Giorgis-Allemand L, Hjertager Krog N, et al. The Urban Exposome during Pregnancy and Its Socioeconomic Determinants. Environmental Health Perspectives. 2018;126(7):077005.
62. Nieuwenhuijsen Mark J, Agier L, Basagaña X, Urquiza J, Tamayo-Uria I, Giorgis-Allemand L, et al. Influence of the Urban Exposome on Birth Weight. Environmental Health Perspectives. 2019;127(4):047007.
63. Smith G, Cirach M, Swart W, Dėdelė A, Gidlow C, van Kempen E, et al. Characterisation of the natural environment: quantitative indicators across Europe. International Journal of Health Geographics. 2017;16(1):16.
64. Beelen R, Hoek G, Vienneau D, Eeftens M, Dimakopoulou K, Pedeli X, et al. Development of NO2 and NOx land use regression models for estimating air pollution exposure in 37 study areas in Europe - the ESCAPE project. Atmos Environ. 2013;72:10 - 23.
65. Aminipouri M, Knudby A, Ho HC. Using multiple disparate data sources to map heat vulnerability: Vancouver case study. Can Geogr. 2016;60(3):356-68.

For peer review only

For peer review only - http://bmjopen.bmj.com/site/about/guidelines.xhtml

BMJ Open

Green CURIOCITY: A study protocol for a European birth cohort study analysing childhood heat-related health impacts and protective effects of urban natural environments.

Journal:	*BMJ Open*
Manuscript ID	bmjopen-2021-052537.R1
Article Type:	Protocol
Date Submitted by the Author:	29-Oct-2021
Complete List of Authors:	van den Bosch, Matilda; Barcelona Institute for Global Health; Universitat Pompeu Fabra<br>Basagaña, Xavier; Barcelona Institute for Global Health; Universitat Pompeu Fabra<br>Mudu, Pierpaolo; World Health Organization European Centre for Environment and Health<br>Kendrovski, Vladimir; World Health Organization European Centre for Environment and Health<br>Maitre, Léa; Barcelona Institute for Global Health<br>Hjertager Krog, Norun; Norwegian Institute of Public Health<br>Aasvang, Gunn Marit; Norwegian Institute of Public Health<br>Grazuleviciene, Regina; Vytauto Didziojo Universitetas<br>McEachan, Rosemary; Bradford Institute for Health Research<br>Vrijheid, Martine; Barcelona Institute for Global Health; Universitat Pompeu Fabra<br>Nieuwenhuijsen, Mark; Barcelona Institute for Global Health; Universitat Pompeu Fabra
**Primary Subject Heading**:	Epidemiology
Secondary Subject Heading:	Public health, Health policy
Keywords:	EPIDEMIOLOGY, PREVENTIVE MEDICINE, PUBLIC HEALTH, Community child health < PAEDIATRICS

SCHOLARONE™
Manuscripts

TITLE PAGE

Title: **Green CURIOCITY: A study protocol for a European birth cohort study analysing childhood heat-related health impacts and protective effects of urban natural environments.**

Authors: van den Bosch, Matilda[1,2,3,4,5], Basagaña, Xavier[1,2,3], Mudu, Pierpaolo[6], Kendrovski, Vladimir[6], Maitre, Léa[1,2,3], Krog, Norun Hjertager[7], Aasvang, Gunn Marit[7], Grazuleviciene, Regina[8], McEachan, Rosemary[9], Vrijheid, Martine[1,2,3], Nieuwenhuijsen, Mark[1,2,3]

Corresponding author: Matilda van den Bosch, Barcelona Institute for Global Health, Doctor Aiguader, 88, 08003 Barcelona, Spain; matilda.vandenbosch@isglobal.org; +34-631827093

[1]ISGlobal, Barcelona, Spain

[2]Universitat Pompeu Fabra (UPF), Barcelona, Spain

[3]CIBER Epidemiología y Salud Pública (CIBERESP), Madrid, Spain

[4]School of Population and Public Health, The University of British Columbia, Vancouver, Canada

[5]Department of Forest and Conservation Sciences, The University of British Columbia, Vancouver, Canada

[6]World Health Organization European Centre for Environment and Health, Bonn, Germany

[7]Norwegian Institute of Public Health, Oslo, Norway

[8]Vytauto Didziojo Universitetas, Kaunas, Lithuania

[9]Bradford Institute for Health Research, Bradford, United Kingdom

Keywords: Childhood health, heat exposure, green spaces, climate change, urban planning

For peer review only

ABSTRACT

Introduction. The European climate is getting warmer and the impact on childhood health and development is insufficiently understood. Equally, how heat related health risks can be reduced through nature-based solutions (NBS), such as exposure to urban natural environments, is unknown. Green CURe In Outdoor CITY spaces (Green CURIOCITY) will analyse how heat exposure during pregnancy affects birth outcomes and how long-term heat exposure may influence children's neurodevelopment. We will also investigate if adverse effects can be mitigated by urban natural environments. A final goal is to visualise intraurban patterns of heat vulnerability and assist planning towards healthier cities.

Methods and analysis. We will use existing data from the Human Early-Life Exposure (HELIX) cohort, which includes information on birth outcomes and neurodevelopment from six European birth cohorts. The cohort is linked to data on prenatal heat exposure and impact on birth outcomes will be analysed with logistic regression models, adjusting for air pollution and noise and socio-behavioural co-variates. Similarly, impact of cumulative and immediate heat exposure on neurodevelopmental outcomes at age five will be assessed. For both analyses, the potentially moderating impact of natural environments will be quantified. For visualisation, Geographic Information Systems (GIS) data will be combined to develop vulnerability maps, demonstrating urban "hot-spots" where the risk of negative impacts of heat is aggravated due to socio-demographic and land use patterns. Finally, geospatial and meteorological data will be used for informing GreenUr, an existing software prototype developed by the WHO Regional Office for Europe to quantify health impacts and augment policy tools for urban green space planning.

Ethics and dissemination. The protocol was approved by the Comité Ético de Investigación Clínica Parc de Salut MAR, Spain. Findings will be published in peer-reviewed journals and presented at policy events. Through stakeholder engagement, the results will also reach user groups and practitioners

For peer review only

STRENGTHS AND LIMITATIONS OF THIS STUDY

- The project will use harmonised data from a large pan-European cohort enabling assessments across different climate zones and
- The accessibility of long-term temporal exposure data facilitates analyses of vulnerability windows
- Analyses of participants who have changed residence over the study period will partly address self-selection bias
- The development of visualisation and policy tools in Green CURIOCITY will provide opportunities for immediate implementation and impact on healthy urban planning
- Some of the cohort contains a disproportionately high number of participants from the highest educational level, potentially limiting the representativeness of the results

For peer review only - http://bmjopen.bmj.com/site/about/guidelines.xhtml

INTRODUCTION

The European climate is getting warmer and the summer of 2019 experienced multiple severe heat waves, shattering all-time temperature records in cities across the continent (1). The morbidity and mortality toll of these events is yet to be unfolded, but we already know the dire effects on a number of vulnerable populations, including more than 20 children who became seriously ill after experiencing temperatures above 40°C in the Netherlands (2). Following climate change, Europe can expect higher average daily temperatures and more frequent heatwaves (3) with severe health and economic consequences (4). The forecast is even grimmer in cities than in rural areas due to the urban heat island effect (5) and long-term effects are insufficiently understood. Heat also aggravates negative impacts of air pollution (6) and intensifies symptoms and mortality risk of many chronic diseases (7, 8). Many countries are unprepared to fully address this challenge, and some populations are at higher risks than others due to, for example, socioeconomic (9), behavioural (10), or demographic factors (11) that reduce resilience. Understanding the full extent of climate change related health impacts across the life course is imperative to accurately act and address the issue, through mitigation, adaption, and treatment options that are available for everyone.

Children are particularly vulnerable to both acute and long-term heat exposure due to a constellation of biological and behavioural factors, including physiology, metabolism, and activity patterns (12, 13). This increases the risk of heat-related morbidity and negative impacts. Recent reviews and research (14, 15) suggests that also prenatal heat exposure has negative effects, possibly because of an increase in oxidative stress and systemic inflammation in response to temperature changes during pregnancy (16, 17). However, additional evidence is clearly needed due to the paucity of studies that have examined the effects of long-term heat exposure on birth outcomes and childhood development. Prior research has largely focused on adults and little is known about how to reduce risks among children. To address this escalating threat, improved evidence and innovative solutions are urgently needed, including insights into risk differences due to sex, socioeconomic status (SES), and living context.

Nature-based solutions (NBS) – defined as "actions which are inspired by, supported by, or copied from nature" designed to address environmental challenges in an adaptive manner while providing economic, social, and environmental benefits (18) – offer unique potential for addressing this threat. The EU Research and Innovation Policy Agenda on NBS and Re-Naturing cities aims to position the EU as a leader in 'innovating with nature' for more resilient societies. In this context, urban natural environments (UNEs, comprising both green spaces such as parks and street trees and blue spaces such as rivers and lakes) stand out as effective NBS to reduce heat-related health risks by cooling via increased shade and evapotranspiration. Existing evidence suggests that the temperature-reducing effect of UNEs ranges from 0.4-3.0°C, or even more, depending on local context (19, 20), with even-larger effects during the night (21). Other, more technical, adaptation strategies exist, but many have proven to be less efficient (22) or energy-intensive (e.g. air condition) (23) consequently escalating greenhouse gas emissions and exacerbating climate change (24). The urgent need to identify more-sustainable solutions (including NBS) is recognised in a forthcoming *Lancet* series on Heat and Health (25).

In parallel, an increasing amount of research indicates numerous childhood health benefits from UNEs, including reduced prevalence of asthma (26), ADHD (27), and overweight (28), and improved birth outcomes (29) and cognitive and other neurodevelopmental functions (30)

For peer review only

with impact across the life course (31). Various pathways for these health benefits have been suggested, but evidence is inconsistent. From this perspective, the mitigating effect of UNEs on heat and subsequent health impacts should be further investigated. As an example, UNEs may contribute to a generally cooler climate, reducing health-related harms of long-term heat exposure. A systematic review from 2016 concluded that the cooling effect of UNEs is one of the more-likely pathways between UNEs and health and that research on this mediator should be prioritised (32).

Knowledge gaps around climate change, childhood health, and Nature Based Solutions

The full impact of the effects of climate change on children's health via acute and long-term heat exposure remains unclear, including differences between climate zones. To identify intra-urban inequalities and areas of greater vulnerability, the roles of specific land-use and socioeconomic factors merit further investigation. The potential of NBS to prevent heat-related health risks must also be analysed in more depth. Although the elderly and chronically ill also represent vulnerable populations, the focus on children is critical from a Developmental Origin of Health and Disease perspective (33), because prenatal and childhood exposures impact health across the life course. Cold-weather events also pose health risks (34), but climate change is more clearly linked to heat-related health risks (35). The research project Green CURe In Outdoor CITY spaces (Green CURIOCITY) will address these knowledge gaps and guide the implementation of sustainable and equitable solutions, including NBS, to address heat exposure and impaired childhood health and development. The topic is of high urgency and relevance because climate change will inevitably increase heat-related morbidity in vulnerable populations, including among preborn and children, and better understanding of NBS could be an approach to reduce current and future risks to health and society.

Aims and Objectives

The objective of Green CURIOCITY is to address the outlined knowledge gaps by analysing the association between pre- and postnatal heat exposure and childhood birth and neurodevelopmental outcomes in different European climate zones. Further on, the aim is to identify whether neighbourhood exposure to UNEs is associated with a lower risk of heat-related adverse health outcomes among children. The goal is also to address implementation by identifying and mapping spatial "hotspots" of childhood heat vulnerability in different European cities with the aim of visualising where interventions, such as NBS, should be prioritised. Finally, the project aims to influence policy making by contributing to further information to a plugin tool, GreenUr, for Geographic Information System (GIS) software (QGIS), developed by the World Health Organization (WHO) Regional Office for Europe. GreenUr measures availability and accessibility of green space and quantifies direct and indirect health impacts at a city scale and Green CURIOCITY aims to specifically contribute to opportunities for quantifying health benefits from urban green spaces within the context of heat reduction.

By the end of Green CURIOCITY, we expect to have identified negative associations between pre- and postnatal heat exposure and childhood birth and neurodevelopmental outcomes. We also expect to find a lower risk of heat-related adverse health outcomes in areas where the abundance of urban natural spaces is high. These results, as well as the visualisation of "hotspots", would have implications for future urban development and also spur research initiatives to address these issues on a global scale.

For peer review only - http://bmjopen.bmj.com/site/about/guidelines.xhtml

METHODS AND ANALYSIS

The inferential studies of Green CURIOCITY are based on linkages of environmental exposure data to cohort data from the Human Early-Life Exposome (HELIX) project (36). HELIX is a collaborative project of six ongoing, longitudinal, population-based birth cohort studies in Europe funded through the EU FP7 Exposome Research programme. Pregnant women in the original cohorts were recruited between 1999 and 2010, and all cohorts included at least one follow-up during the prenatal period, at birth, and after delivery. The complete HELIX study population includes 31,472 mother-child pairs at the time of delivery and all information is stored in a common data warehouse. The harmonisation process of data from the national cohorts in HELIX followed expert input for standardisation rules and was based on a coding-system, subsequent to checking and matching of cohort-specific variables (36).

Study area and national cohorts

The birth cohorts of HELIX include the Born in Bradford (BiB) study in the UK (37), the Étude des Déterminants pré et postnatals du dévelopment et de la santé de l'Enfant (EDEN) study in France (38), the INfancia y Medio Ambiente (INMA) cohort in Spain (39), the Kaunus cohort (KANC) in Lithuania (40), the Norwegian Mother, Father and Child Cohort Study (MoBa) (41), and the RHEA Mother Child Cohort study in Crete, Greece (42) (Table 1).

Table 1 Summary of the national cohorts included in HELIX. Adapted from Maitre et al. (2018) (36)

Cohort	Years of birth	Region covered by HELIX	No. of births in HELIX entire cohort
**BiB, UK**	2007-2010	Bradford	10,849
**EDEN, France**	2003-2006	Nancy and Poitiers, urban areas	1,900
**INMA, Spain**	2003-2008	Gipuzkoa, Sabadell, Valencia	2,063
**KANC, Lithuania**	2007-2008	Kaunas	4,107
**MoBa, Norway**	1999-2008	Oslo	11,095
**RHEA, Greece**	2007-2008	Heraklion	1,458
**Total**			**31,472**

BiB: Born in Bradford; EDEN: Étude des Déterminants pré et postnatals du development et de la santé de l'Enfant; INMA: INfancia y Medio Ambiente; KANC: Kaunas cohort; MoBa: Norwegian Mother, Father and Child Cohort Study.

Primary outcomes in Green CURIOCITY: Birth outcome data are available for the entire cohort (n=31,472) and include gestational duration, birth weight and length, and size for gestational age. Exclusion criteria in most of the cohorts were twin pregnancies, women with diabetes or pregnancy induced hypertension, and mothers under 16 years of age.

The availability of neurodevelopmental indicators varies across the cohorts, but data have been harmonised for cognitive function (verbal and non-verbal) and for psychomotor function (fine and gross motor function). In total 11,423 children have harmonised data for cognitive and/or psychomotor function linked to environmental exposures. Not all cohorts collected data for all the different outcomes (Table 2). KANC did not collect data on neurodevelopmental outcomes.

For peer review only - http://bmjopen.bmj.com/site/about/guidelines.xhtml

Table 2. Harmonised neurodevelopmental outcome data per cohort to be used in Green CURIOCITY. (N=11,423)

Neurodevelopmental outcome	BiB	EDEN	INMA	MoBa	RHEA	Subjects in harmonised dataset
**Cognition function**	0	1,100	1,420	0	779	3,299
**Verbal cognitive function**	2,057	1,107	1,420	5,834	779	11,197
**Non-verbal cognitive function**	0	1,107	1,420	0	779	3,306
**Psychomotor function**	0	0	1,420	5,939	779	8,138
**Gross motor function**	0	0	1,420	5,864	779	8,112
**Fine motor function**	1,909	1,120	1,420	5,913	779	11,092

All outcome variables have been harmonised and standardised to a mean of 100 and a standard deviation of 15, making them comparable across the cohorts. The tests that were used for various neurodevelopmental outcomes in the respective cohorts are listed in table 3. All tests were evaluated at the age of 4.5 years.

Table 3. The respective tests and scales used in the separate cohorts before harmonisation.

	**Cognitive function**			**Psychomotor function**		
	Cognitive function	Verbal cognition	Non-verbal cognition	Psychomotor function	Gross motor	Fine motor
**BiB, UK**						
British Picture Vocabulary Scale (43)		x				
Clinical Kinematic Assessment Tool (44)						x
**EDEN, France**						
Weschler Preschool and Primary Scale of Intelligence (45)	x	x	x			
Peg moving task (46)						x
**INMA, Spain**						
McCarthy Scales of Children's Abilities (47)	X	X	X	X	X	X
**MoBa, Norway**						
Child Development Inventory (48)		X		X	X	X
**RHEA, Greece**						
McCarthy Scales of Children's Abilities (47)	x	x	x	x	x	x

Co-variates

The interactions between environmental exposures, childhood health outcomes and other social, biological, and cultural variables are complex and the evidence of associations is often inconsistent. The final selection of confounder variables to include in the analyses will be determined a priori based on existing evidence and by using a Directed Acyclic Graphs (DAG) approach. HELIX contains extensive information on a number of key covariates and based on a preliminary literature search on potential confounders of the association between prenatal heat exposure and birth outcomes, we will consider including child sex, season of birth (49,

For peer review only - http://bmjopen.bmj.com/site/about/guidelines.xhtml

50), urban living during pregnancy (51), maternal age and education (52, 53), maternal body mass index (BMI) (54), parental country of origin (55), maternal health behaviour (such as smoking and alcohol intake) during pregnancy (56), and individual and area level socioeconomic deprivation indicators (54). The analysis of neurodevelopmental outcomes will consider the same confounders and, in addition, maternal cognitive function (57) and attendance to nursery school (58).

Environmental exposure variables

Environmental exposure data for Green CURIOCITY exist in an integrated Geographic Information System (GIS) environment, contained in the HELIX database. Using complete residential address histories, exposure estimates per trimester during the prenatal period and average annual values up to age of neurodevelopmental assessment have been assigned to the participants.

By the time of submission and approval of this proposal, a heat index was planned to be developed based on data from local meteorological stations contained within the HELIX GIS-environment, including (1) temperature (average of mean/max/min during pregnancy period, per trimester, and at birth); (3) relative humidity (average of mean/max/min during pregnancy period, per trimester, and at birth); and (4) atmospheric pressure during pregnancy and per trimester. The aim was to also include land surface temperature (LST). Based on development of the HELIX GIS-environment, this project now aims to use a heat index derived from the E-OBS dataset within the Copernicus Programme (https://climate.copernicus.eu/). This index is derived using daily minimum and maximum temperature, daily precipitation sum, global radiation, and relative humidity and the spatial resolution is 0.1º (approximately 11 km). The values are calculated annually, monthly, and seasonally. To address specific time windows of potential vulnerability, we will also test, in separate models, impact of temperature, relative humidity and atmospheric pressure, using the time specific data contained in the HELIX dataset. The meteorological values per trimester will be linked to data on birth outcomes and the opportunity for assessing specific heat waves will be explored. Impact on neurodevelopment will be assessed by linking the heat index value prenatally as well as postnatal annual values up to the age of outcome assessment.

To assess the modifying impact of urban natural spaces on the association between heat exposure and childhood health outcomes, we will use data from the European Environmental Agency's 'Urban Atlas' (year 2006) (59), apart from for the Norwegian cohort (MoBa) where a similar local land cover map, Norwegian map, N50, was used (60). The Urban Atlas contains 17 land use classes and is developed from image classification and interpretation of high-resolution satellite imagery. The accuracy level is approximately 85% for artificial surfaces. Across European cities, it provides harmonised information of land use, including, for example, green urban areas, water, sport and recreational facilities, impervious surfaces, and built-up areas. We assume relatively minor changes in urban green space land cover over the study period. Apart from exposure values, we will use area and distance to nearest green and blue spaces, as of previous protocols (61, 62). We will also use a general vegetation indicator, the Normalized Difference Vegetation Index (NDVI) (63), derived from 30m spatial resolution Landsat images and provided in raster maps.. To achieve maximum exposure contrast, the NDVI values that are included in the HELIX GIS-environment are based on available cloud-free Landsat TM images during the period between May and August for years relevant to our period of study. Greenness values are calculated and averaged within 100-, 300-, and 500-m buffers around each residential address. These buffer zones are commonly used in

For peer review only - http://bmjopen.bmj.com/site/about/guidelines.xhtml

epidemiological studies assessing the impact of natural spaces on health outcomes. In general, the 300m buffer zone is considered as most indicative of home exposure (64), but additional buffer zones will also be tested for sensitivity analyses. The project will adjust for air pollution exposure (NO_2, NOx, PM_{10}, $PM_{2.5}$) available from existing land use regression (LUR) models developed in the European Study of Cohorts for Air Pollution Effects (ESCAPE) project. See Beelen et al. (65) and Eeftens et al. (66) for further information about the LUR models. The values are extrapolated back in time, using data from existing regulatory monitors, and averaged per trimester, pregnancy period, birth, and annually. In addition, we will control for noise exposure, available from European road traffic noise maps from the European Environment Agency with continuous and categorical values (L_{den}).

Statistical analysis
We will use logistic regression to estimate the association between trimester-specific temperature and risk of short gestational duration or small for gestational age and linear regression to estimate the association between trimester-specific temperature and term birth weight, standardized via z-scores and adjusted for key co-variates. Results will be pooled overall and by geographic regions and climate zones (Mediterranean: Spain and Greece; Temperate: UK and France; Boreal: Norway). The pooling per climate zone will contribute to the understanding of potential differences in the adaptation to heat as well as in the modifying effect of natural spaces on the selected outcomes. In addition, stratified analyses will be conducted to assess moderation by sex and SES. We will develop structural equation models (SEMs) to explore the potential for UNEs to mitigate adverse heat-related effects on birth outcomes. SEM analyses will allow us to test the statistical significance of a mediation effect (i.e., that improved birth outcomes occur via cooling effects of UNEs), while adjusting for confounding and neighbourhood dependency. Using SEM will also allow us to detect indirect effects (i.e., that UNEs reduce harmful effects of heat even if direct heat-related effects on birth outcomes are not detected). Our primary approach will be to use a causal mediation analysis framework, as suggested by Imai et al. (67), but a detailed statistical plan will be developed as the project proceeds and alternative approaches, such as partial least squares-SEMs (PLS-SEM) (68) will also be considered. Results will be pooled by climate zone and we will run sensitivity analyses adjusting for air pollution and noise. We will also develop logistic regression models to test the association between long-term heat exposure and neurodevelopment, adjusting for key confounders. We will apply time-weighted averages across the full exposure period, pre- and postnatally, carrying out sub-analyses among children who have moved to assess the impact of exposure length and to partially address self-selection bias. Additional analyses will be stratified by sex and SES and pooled by geographic region and climate zone. We will use SEM with a similar approach and rationale as for the birth outcome analyses, assessing the mitigating effect of UNE exposure, as well as similar sensitivity analyses.

Mapping and spatial overlay design
For the "visualisation" study of Green CURIOCITY we will conduct a spatial overlay analysis to identify areas where different environmental (e.g. low amount of UNEs) or demographic (e.g. low age or low income) vulnerability factors coincide with high temperatures, so called urban "hotspots". We will assess the approach and consider different methods based on spatial clustering, such as LISA or multivariate clustering (69, 70). Geospatial data capturing the intraurban spatial distribution of environmental (heat, UNE, built environment, and impervious surfaces) vulnerability indicators will be downloaded from the HELIX GIS environment and

For peer review only - http://bmjopen.bmj.com/site/about/guidelines.xhtml

demographic data (age group, sex, and SES) from municipal resources. Environmental data will be averaged per the spatial unit that is aligned with SES data aggregation (e.g. dissemination areas) in respective cities. We primarily aim to use a simple combination overlay analysis, but more complex methods, such as union, intersection, or identity operations may also be considered. Subsequently, we will apply a ranking method and identify the most-vulnerable areas, based on prevalence of vulnerability factors, using a cut-off of 25%, similar to the approach used by Aminipouri et al. (2016) (71). The 25% threshold is an easily interpretable number, which makes the approach useful for city officials to replicate for making decisions about directing investments in NBS interventions.

Software expansion

For the implementation tool, expansion of an existing software prototype developed for QGIS by WHO (GreenUr, https://www.euro.who.int/en/health-topics/environment-and-health/urban-health/activities/greenur-the-green-urban-spaces-and-health-tool) will be developed based on adaptation of existing algorithms and modelled quantification of heat related health effects following changes in UNEs. Raster (Landsat) and vector data (Urban Atlas) of UNEs in combination with meteorological input data will be incorporated in the models. GreenUr is a prototype under development and the exact input data and what functions to incorporate will depend on the general evolvement of Green CURIOCITY and on the current processing of the prototype.

PATIENT AND PUBLIC INVOLVEMENT Neither patients nor the public were involved in the development of this protocol. Patient and public involvement were, however, part of the wider HELIX project per each cohort.

ETHICS AND DISSEMINATION

All data to be used in Green CURIOCITY are available from the HELIX cohort. The six national cohorts on which HELIX are based had undergone ethical review and approval at initiation and new approvals were acquired for the work in HELIX. An additional evaluation and ethics approval has been acquired for Green CUROICTY project from the Comité Ético de investigación Clínica Parc de Salut MAR, Spain (reference number 2020/9567/I). The data are pseudonymised and all procedures of Green CURIOCITY that will include analyses of study participants will follow the ethics guidelines of ISGlobal's Ethics Committee and the HELIX Ethics Task Force (36). Data will be safeguarded to protect the confidentiality and anonymity of the participants and is consistent with the terms of the consent signed by participants in the respective national cohorts. Adequate measures to ensure health data protection and confidentiality will be taken, according to European directive on the protection of individuals with regard to the processing of personal data and free movement of such data (2016/679, General Data Protection Regulation, GDPR). All reported results will pertain to analyses of aggregate data; no variables or combination of variables that can identify an individual will be associated with any published or unpublished report of the study.

Results from Green CURIOCITY will be disseminated through high-impact scientific journals, conference presentations, expert seminars, and policy briefs. The project has connections to broad international, interdisciplinary networks, such as the International Union for Conservation of Nature and the World Health Organization (WHO), which will support dissemination to a global audience. Methods and results will also be communicated through research briefs and reports and via institutional social media platforms and project websites.

For peer review only - http://bmjopen.bmj.com/site/about/guidelines.xhtml

There are plans for roundtable events with urban planners and key decision-makers to share results of the vulnerability-mapping exercises and to integrate findings into sustainable urban plans, although these strategies may have to be adapted due to the current COVID-19 situation. In addition, the project aims to prepare educational material for distribution at schools, health care clinics, and public health websites, including infographics and worksheets describing how heat impacts children, summarizing recommendations regarding "green" schoolyards and playgrounds, and highlighting the importance of heat prevention during pregnancy.

AUTHORS' CONTRIBUTIONS

MvdB drafted this manuscript on the basis of a grant proposal that was devised and written by MvdB in collaboration with MN, XB, PM, and VK. MV is principal investigator of HELIX. LM coordinated the collection and harmonisation of the entire HELIX database. GMA, RG, RM co-ordinated HELIX data collection and research protocols in respective national cohorts. NHK provided environmental data for MoBa. All authors contributed to drafting, editing, and reviewing the manuscript. All authors read the final version of this manuscript, approved it for submission for publication, and agreed to be accountable for all aspects of the work. The proposal was approved for funding on 31/03/2020.

FUNDING STATEMENT

This work is supported by the European Commission through a Horizon 2020 Marie Skłodowska-Curie Grant, Reference number 891538.

LM is funded by a Juan de la Cierva-Incorporación fellowship (IJC2018-035394-I) awarded by the Spanish Ministerio de Economía, Industria y Competitividad.

COMPETING INTERESTS STATEMENT

Nothing to declare.

PM and VK are staff members of the WHO. The authors alone are responsible for the views expressed in this publication and they do not necessarily represent the decisions or stated policy of the WHO.

REFERENCES

1. Mitchell D, Kornhuber K, Huntingford C, Uhe P. The day the 2003 European heatwave record was broken. The Lancet Planetary Health. 2019;3(7):e290-e2.
2. APNews. The Latest: Heat wave smashes weather records in Europe 2019 [Available from: https://www.apnews.com/e29811558e0344e6ae0f326dadfab2e1.
3. Meehl GA, Tebaldi C. More intense, more frequent, and longer lasting heat waves in the 21st century. Science. 2004;305.
4. Peng RD, Bobb JF, Tebaldi C, McDaniel L, Bell ML, Dominici F. Toward a quantitative estimate of future heat wave mortality under global climate change. Environ Health Perspect. 2011;119(5):701-6.
5. Oke TR. City size and the urban heat island. Atmospheric Environment. 1973;7(8):769-79.

For peer review only - http://bmjopen.bmj.com/site/about/guidelines.xhtml

6. McCormack MC, Belli AJ, Waugh D, Matsui EC, Peng RD, Williams DL, et al. Respiratory effects of indoor heat and the interaction with air pollution in chronic obstructive pulmonary disease. Annals of the American Thoracic Society. 2016;13(12):2125-31.
7. Watts N, Amann M, Arnell N, Ayeb-Karlsson S, Belesova K, Berry H, et al. The 2018 report of the Lancet Countdown on health and climate change: shaping the health of nations for centuries to come. The Lancet. 2018;392(10163):2479-514.
8. Xu R, Zhao Q, Coelho MSZS, SaldivaPaulo H.N., Zoungas S, Huxley RR, et al. Association between Heat Exposure and Hospitalization for Diabetes in Brazil during 2000-2015: A Nationwide Case-Crossover Study. Environmental Health Perspectives. 2019;127(11):117005.
9. Reid CE, O'Neill MS, Gronlund CJ, Brines SJ, Brown DG, Diez-Roux AV, et al. Mapping community determinants of heat vulnerability. Environ Health Perspect. 2009;117(11):1730-6.
10. Johnson DP, Stanforth A, Lulla V, Luber G. Developing an applied extreme heat vulnerability index utilizing socioeconomic and environmental data. Applied Geography. 2012;35(1-2):23-31.
11. Díaz J, Carmona R, Mirón I, Ortiz C, León I, Linares C. Geographical variation in relative risks associated with heat: update of Spain's Heat Wave Prevention Plan. Environment international. 2015;85:273-83.
12. Xu Z, Etzel RA, Su H, Huang C, Guo Y, Tong S. Impact of ambient temperature on children's health: A systematic review. Environmental Research. 2012;117:120-31.
13. Xu Z, Sheffield PE, Su H, Wang X, Bi Y, Tong S. The impact of heat waves on children's health: a systematic review. International Journal of Biometeorology. 2014;58(2):239-47.
14. Zhang Y, Yu C, Wang L. Temperature exposure during pregnancy and birth outcomes: An updated systematic review of epidemiological evidence. Environmental Pollution. 2017;225:700-12.
15. Spolter F, Kloog I, Dorman M, Novack L, Erez O, Raz R. Prenatal exposure to ambient air temperature and risk of early delivery. Environ Int. 2020;142:105824.
16. Halonen JI, Zanobetti A, Sparrow D, Vokonas PS, Schwartz J. Associations between outdoor temperature and markers of inflammation: a cohort study. Environmental Health. 2010;9(1):42.
17. Ferguson KK, Kamai EM, Cantonwine DE, Mukherjee B, Meeker JD, McElrath TF. Associations between repeated ultrasound measures of fetal growth and biomarkers of maternal oxidative stress and inflammation in pregnancy. American Journal of Reproductive Immunology. 2018;80(4):e13017.
18. ECDG. Towards an EU Research and Innovation Policy Agenda for Nature-Based Solutions & Re-Naturing Cities Final Report of the Horizon 2020 Expert Group on "Nature-Based Solutions and Re-Naturing Cities". Luxembourg; 2015.
19. TNC. Planting healthy air: A global analysis of the role of urban trees in addressing particulate matter pollution and extreme heat. The Nature Conservancy; 2016.
20. Venter ZS, Krog NH, Barton DN. Linking green infrastructure to urban heat and human health risk mitigation in Oslo, Norway. Science of The Total Environment. 2020;709:136193.
21. Fryd O, Pauleit S, Bühler O. The role of urban green space and trees in relation to climate change. CAB Reviews: Perspectives in Agriculture, Veterinary Science, Nutrition and Natural Resources. 2011;6:1-18.
22. Weinberger KR, Zanobetti A, Schwartz J, Wellenius GA. Effectiveness of National Weather Service heat alerts in preventing mortality in 20 US cities. Environment International. 2018;116:30-8.
23. Davis LW, Gertler PJ. Contribution of air conditioning adoption to future energy use under global warming. Proceedings of the National Academy of Sciences. 2015;112(19):5962-7.
24. Abel DW, Holloway T, Harkey M, Meier P, Ahl D, Limaye VS, et al. Air-quality-related health impacts from climate change and from adaptation of cooling demand for buildings in the eastern United States: An interdisciplinary modeling study. PLoS Medicine. 2018;15(7).

For peer review only - http://bmjopen.bmj.com/site/about/guidelines.xhtml

25. Capon A, Jay O, Ebi K, Lo S. Heat and health: a forthcoming Lancet Series. The Lancet. 2019;394(10198):551-2.
26. Sbihi H, Tamburic L, Koehoorn M, Brauer M. Greenness and incident childhood asthma: A 10-year follow-up in a population-based birth cohort. American Journal of Respiratory and Critical Care Medicine. 2015;192(9):1131-3.
27. Donovan GH, Michael YL, Gatziolis D, Mannetje At, Douwes J. Association between exposure to the natural environment, rurality, and attention-deficit hyperactivity disorder in children in New Zealand: a linkage study. The Lancet Planetary Health. 2019;3(5):e226-e34.
28. Bao W-W, Yang B-Y, Zou Z-Y, Ma J, Jing J, Wang H-J, et al. Greenness surrounding schools and adiposity in children and adolescents: Findings from a national population-based study in China. Environmental Research. 2021;192:110289.
29. Akaraci S, Feng X, Suesse T, Jalaludin B, Astell-Burt T. A Systematic Review and Meta-Analysis of Associations between Green and Blue Spaces and Birth Outcomes. International journal of environmental research and public health. 2020;17(8):2949.
30. Mygind L, Kurtzhals M, Nowell C, Melby PS, Stevenson MP, Nieuwenhuijsen M, et al. Landscapes of becoming social: A systematic review of evidence for associations and pathways between interactions with nature and socioemotional development in children. Environment International. 2021;146:106238.
31. Browning MHEM, Rigolon A. Could nature help children rise out of poverty? Green space and future earnings from a cohort in ten U.S. cities. Environmental Research. 2019.
32. **van den Bosch** M, Ode Sang A. Urban natural environments as nature-based solutions for improved public health - A systematic review of reviews. Environ Res. 2017;158:373-84.
33. Gluckman P, Hanson M. The developmental origins of health and disease: An overview. Developmental Origins of Health and Disease2006. p. 1-5.
34. Gasparrini A, Guo Y, Hashizume M, Lavigne E, Zanobetti A, Schwartz J. Mortality risk attributable to high and low ambient temperature: a multicountry observational study. Lancet. 2015;386.
35. Gasparrini A, Guo Y, Sera F, Vicedo-Cabrera AM, Huber V, Tong S, et al. Projections of temperature-related excess mortality under climate change scenarios. The Lancet Planetary Health. 2017.
36. Maitre L, de Bont J, Casas M, Robinson O, Aasvang GM, Agier L, et al. Human Early Life Exposome (HELIX) study: a European population-based exposome cohort. BMJ Open. 2018;8(9):e021311.
37. Wright J, Small N, Raynor P, Tuffnell D, Bhopal R, Cameron N, et al. Cohort Profile: The Born in Bradford multi-ethnic family cohort study. International Journal of Epidemiology. 2012;42(4):978-91.
38. Heude B, Forhan A, Slama R, Douhaud L, Bedel S, Saurel-Cubizolles M-J, et al. Cohort Profile: The EDEN mother-child cohort on the prenatal and early postnatal determinants of child health and development. International Journal of Epidemiology. 2015;45(2):353-63.
39. Guxens M, Ballester F, Espada M, Fernández MF, Grimalt JO, Ibarluzea J, et al. Cohort profile: the INMA—INfancia y Medio Ambiente—(environment and childhood) project. International journal of epidemiology. 2012;41(4):930-40.
40. Grazuleviciene R, Danileviciute A, Nadisauskiene R, Vencloviene J. Maternal smoking, GSTM1 and GSTT1 polymorphism and susceptibility to adverse pregnancy outcomes. International journal of environmental research and public health. 2009;6(3):1282-97.
41. Magnus P, Birke C, Vejrup K, Haugan A, Alsaker E, Daltveit AK, et al. Cohort profile update: the Norwegian mother and child cohort study (MoBa). International journal of epidemiology. 2016;45(2):382-8.
42. Chatzi L, Leventakou V, Vafeiadi M, Koutra K, Roumeliotaki T, Chalkiadaki G, et al. Cohort profile: the mother-child cohort in Crete, Greece (Rhea study). International journal of epidemiology. 2017;46(5):1392-3k.

For peer review only - http://bmjopen.bmj.com/site/about/guidelines.xhtml

43. Dunn LM, Dunn DM. The British picture vocabulary scale: GL Assessment Limited; 2009.
44. Culmer PR, Levesley MC, Mon-Williams M, Williams JH. A new tool for assessing human movement: the Kinematic Assessment Tool. J Neurosci Methods. 2009;184(1):184-92.
45. Warschausky S. Wechsler Preschool and Primary Scale of Intelligence. In: Kreutzer JS, DeLuca J, Caplan B, editors. Encyclopedia of Clinical Neuropsychology. New York, NY: Springer New York; 2011. p. 2690-3.
46. Annett M. Handedness and Braisn Assymetry. 1 ed. London: Psychology Press; 2001.
47. Levin E. McCarthy Scales of Children's Abilities. In: Goldstein S, Naglieri JA, editors. Encyclopedia of Child Behavior and Development. Boston, MA: Springer US; 2011. p. 928-9.
48. Ireton H, Glascoe FP. Assessing children's development using parents' reports. The Child Development Inventory. Clin Pediatr (Phila). 1995;34(5):248-55.
49. Grootendorst-Van Mil NH, Steegers-Theunissen RPM, Hofman A, Jaddoe VWV, Verhulst FC, Tiemeier H. Brighter children? The association between seasonality of birth and child IQ in a population-based birth cohort. BMJ Open. 2017;7(2).
50. Hemati Z, Keikha M, Riahi R, Daniali SS, Goudarzi M, Kelishadi R. A systematic review on the association of month and season of birth with future anthropometric measures. Pediatric Research. 2020.
51. Skrivankova V, Zwahlen M, Adams M, Low N, Kuehni C, Egger M. Spatial epidemiology of gestational age and birth weight in Switzerland: Census-based linkage study. BMJ Open. 2019;9(10).
52. Shapiro GD, Bushnik T, Wilkins R, Kramer MS, Kaufman JS, Sheppard AJ, et al. Adverse birth outcomes in relation to maternal marital and cohabitation status in Canada. Annals of Epidemiology. 2018;28(8):503-9.e11.
53. Weng YH, Yang CY, Chiu YW. Risk assessment of adverse birth outcomes in relation to maternal age. PLoS ONE. 2014;9(12).
54. Clayborne ZM, Giesbrecht GF, Bell RC, Tomfohr-Madsen LM. Relations between neighbourhood socioeconomic status and birth outcomes are mediated by maternal weight. Social Science and Medicine. 2017;175:143-51.
55. Komro KA, Markowitz S, Livingston MD, Wagenaar AC. Effects of State-Level Earned Income Tax Credit Laws on Birth Outcomes by Race and Ethnicity. Health Equity. 2019;3(1):61-7.
56. Bird AL, Grant CC, Bandara DK, Mohal J, Atatoa-Carr P, Wise MR, et al. Maternal health in pregnancy and associations with adverse birth outcomes: Evidence from Growing Up in New Zealand. Aust New Zealand J Obstet Gynaecol. 2017;57(1):16-24.
57. Ronfani L, Vecchi Brumatti L, Mariuz M, Tognin V, Bin M, Ferluga V, et al. The complex interaction between home environment, socioeconomic status, maternal IQ and early child neurocognitive development: A multivariate analysis of data collected in a newborn cohort study. PLoS ONE. 2015;10(5).
58. Barnett WS. Long-Term Effects of Early Childhood Programs on Cognitive and School Outcomes. The Future of Children. 1995;5(3):25-50.
59. European_Union. Mapping Guide for a European Urban Atlas. European Environment Agency, EEA, Copenhagen, Denmark; 2011.
60. https://kartkatalog.geonorge.no/metadata/n50-map-data/ea192681-d039-42ec-b1bc-f3ce04c189ac. [
61. Robinson O, Tamayo I, de Castro M, Valentin A, Giorgis-Allemand L, Hjertager Krog N, et al. The Urban Exposome during Pregnancy and Its Socioeconomic Determinants. Environmental Health Perspectives. 2018;126(7):077005.
62. Nieuwenhuijsen Mark J, Agier L, Basagaña X, Urquiza J, Tamayo-Uria I, Giorgis-Allemand L, et al. Influence of the Urban Exposome on Birth Weight. Environmental Health Perspectives. 2019;127(4):047007.

For peer review only - http://bmjopen.bmj.com/site/about/guidelines.xhtml

63. Smith G, Cirach M, Swart W, Dėdelė A, Gidlow C, van Kempen E, et al. Characterisation of the natural environment: quantitative indicators across Europe. International Journal of Health Geographics. 2017;16(1):16.
64. **Annerstedt van den Bosch** M, Mudu P, Uscila V, Barrdahl M, Kulinkina A, Staatsen B, et al. Development of an urban green space indicator and the public health rationale. Scandinavian Journal of Public Health. 2016;44:159-67.
65. Beelen R, Hoek G, Vienneau D, Eeftens M, Dimakopoulou K, Pedeli X, et al. Development of NO2 and NOx land use regression models for estimating air pollution exposure in 37 study areas in Europe - the ESCAPE project. Atmos Environ. 2013;72:10 - 23.
66. Eeftens M, Beelen R, de Hoogh K, Bellander T, Cesaroni G, Cirach M, et al. Development of Land Use Regression Models for PM2.5, PM2.5 Absorbance, PM10 and PMcoarse in 20 European Study Areas; Results of the ESCAPE Project. Environmental Science & Technology. 2012;46(20):11195-205.
67. Imai K, Keele L, Tingley D. A general approach to causal mediation analysis. Psychological Methods. 2010;15(4):309-34.
68. Astrachan CB, Patel VK, Wanzenried G. A comparative study of CB-SEM and PLS-SEM for theory development in family firm research. Journal of Family Business Strategy. 2014;5(1):116-28.
69. Anselin L. Local Indicators of Spatial Association—LISA. Geographical Analysis. 1995;27(2):93-115.
70. Peeters A, Zude M, Käthner J, Ünlü M, Kanber R, Hetzroni A, et al. Getis–Ord's hot- and cold-spot statistics as a basis for multivariate spatial clustering of orchard tree data. Computers and Electronics in Agriculture. 2015;111:140-50.
71. Aminipouri M, Knudby A, Ho HC. Using multiple disparate data sources to map heat vulnerability: Vancouver case study. Can Geogr. 2016;60(3):356-68.

For peer review only

For peer review only - http://bmjopen.bmj.com/site/about/guidelines.xhtml
